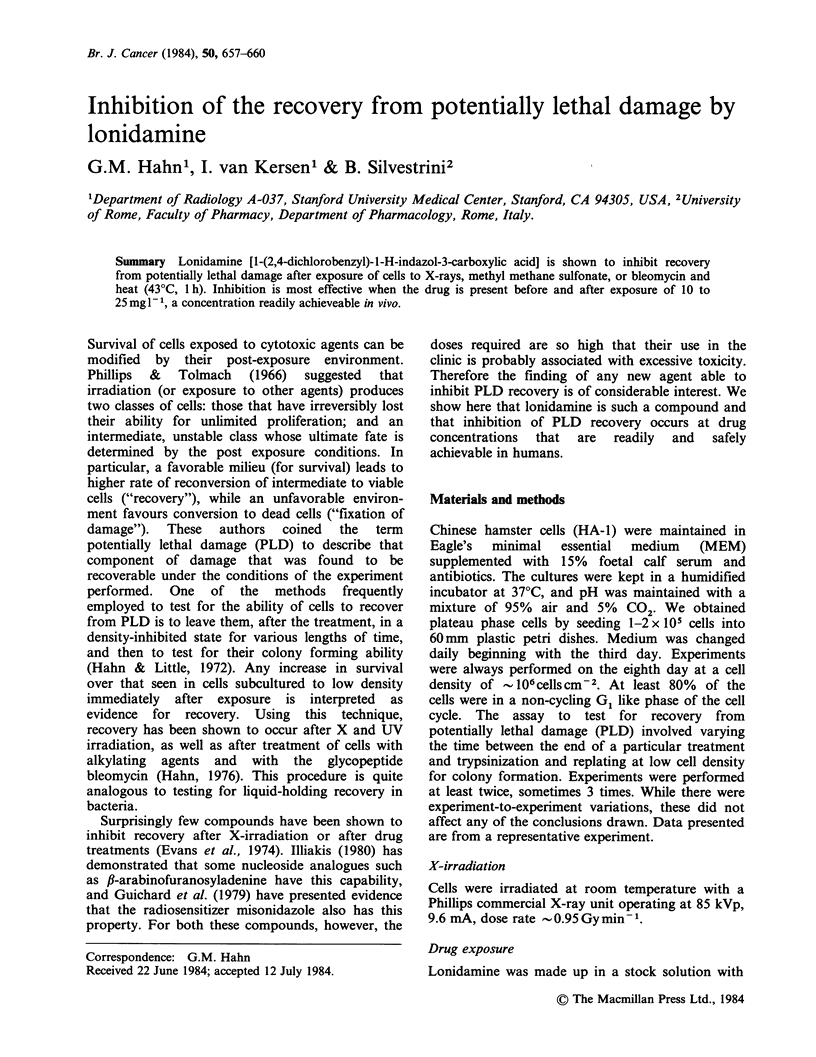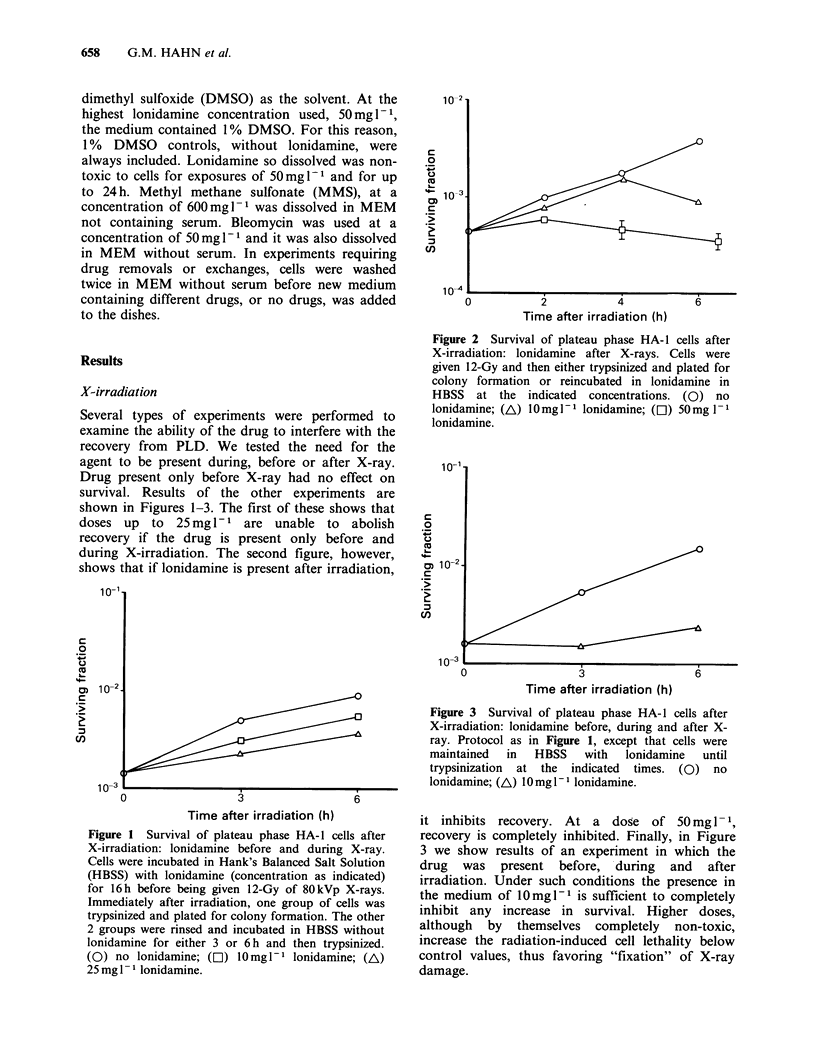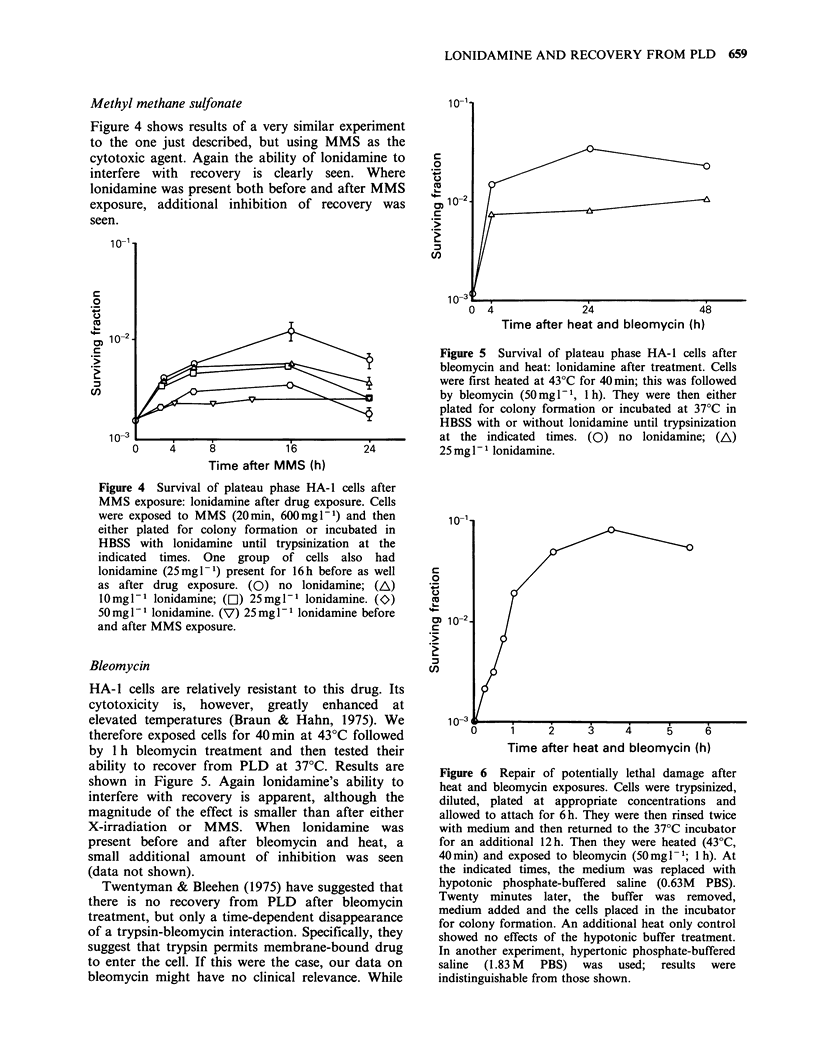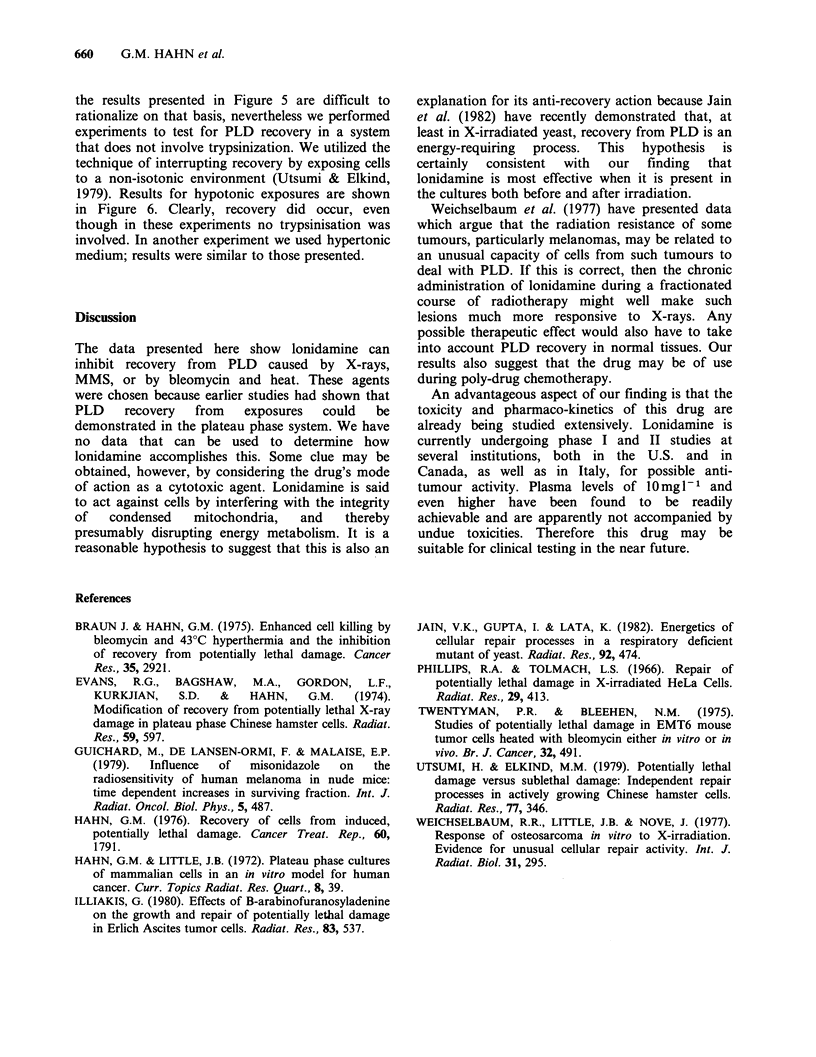# Inhibition of the recovery from potentially lethal damage by lonidamine.

**DOI:** 10.1038/bjc.1984.232

**Published:** 1984-11

**Authors:** G. M. Hahn, I. van Kersen, B. Silvestrini

## Abstract

Lonidamine [1-(2,4-dichlorobenzyl)-1-H-indazol-3-carboxylic acid] is shown to inhibit recovery from potentially lethal damage after exposure of cells to X-rays, methyl methane sulfonate, or bleomycin and heat (43 degrees C, 1h). Inhibition is most effective when the drug is present before and after exposure of 10 to 25 mg l-1, a concentration readily achievable in vivo.


*Br. J. Cancer* (1984), **50**, 657–660

### Inhibition of the recovery from potentially lethal damage by lonidamine

G.M. Hahn[1], I. van Kersen[1] & B. Silvestrini[2]

[1]*Department of Radiology A-037, Stanford University Medical Center, Stanford, CA 94305, USA,* [2]*University of Rome, Faculty of Pharmacy, Department of Pharmacology, Rome, Italy.*

**Summary** Lonidamine [1-(2,4-dichlorobenzyl)-1-H-indazol-3-carboxylic acid] is shown to inhibit recovery from potentially lethal damage after exposure of cells to X-rays, methyl methane sulfonate, or bleomycin and heat (43°C, 1 h). Inhibition is most effective when the drug is present before and after exposure of 10 to $25\,mg\,l^{-1}$, a concentration readily achieveable *in vivo*.

Survival of cells exposed to cytotoxic agents can be modified by their post-exposure environment. Phillips & Tolmach (1966) suggested that irradiation (or exposure to other agents) produces two classes of cells: those that have irreversibly lost their ability for unlimited proliferation; and an intermediate, unstable class whose ultimate fate is determined by the post exposure conditions. In particular, a favorable milieu (for survival) leads to higher rate of reconversion of intermediate to viable cells ("recovery"), while an unfavorable environment favours conversion to dead cells ("fixation of damage"). These authors coined the term potentially lethal damage (PLD) to describe that component of damage that was found to be recoverable under the conditions of the experiment performed. One of the methods frequently employed to test for the ability of cells to recover from PLD is to leave them, after the treatment, in a density-inhibited state for various lengths of time, and then to test for their colony forming ability (Hahn & Little, 1972). Any increase in survival over that seen in cells subcultured to low density immediately after exposure is interpreted as evidence for recovery. Using this technique, recovery has been shown to occur after X and UV irradiation, as well as after treatment of cells with alkylating agents and with the glycopeptide bleomycin (Hahn, 1976). This procedure is quite analogous to testing for liquid-holding recovery in bacteria.

Surprisingly few compounds have been shown to inhibit recovery after X-irradiation or after drug treatments (Evans *et al.,* 1974). Illiakis (1980) has demonstrated that some nucleoside analogues such as $\beta$-arabinofuranosyladenine have this capability, and Guichard *et al.* (1979) have presented evidence that the radiosensitizer misonidazole also has this property. For both these compounds, however, the doses required are so high that their use in the clinic is probably associated with excessive toxicity. Therefore the finding of any new agent able to inhibit PLD recovery is of considerable interest. We show here that lonidamine is such a compound and that inhibition of PLD recovery occurs at drug concentrations that are readily and safely achievable in humans.

Correspondence: G.M. Hahn
Received 22 June 1984; accepted 12 July 1984.

### Materials and methods

Chinese hamster cells (HA-1) were maintained in Eagle's minimal essential medium (MEM) supplemented with 15% foetal calf serum and antibiotics. The cultures were kept in a humidified incubator at 37°C, and pH was maintained with a mixture of 95% air and 5% $CO_2$. We obtained plateau phase cells by seeding $1–2\times10^5$ cells into 60 mm plastic petri dishes. Medium was changed daily beginning with the third day. Experiments were always performed on the eighth day at a cell density of $\sim10^6\,cells\,cm^{-2}$. At least 80% of the cells were in a non-cycling $G_1$ like phase of the cell cycle. The assay to test for recovery from potentially lethal damage (PLD) involved varying the time between the end of a particular treatment and trypsinization and replating at low cell density for colony formation. Experiments were performed at least twice, sometimes 3 times. While there were experiment-to-experiment variations, these did not affect any of the conclusions drawn. Data presented are from a representative experiment.

### *X-irradiation*

Cells were irradiated at room temperature with a Phillips commercial X-ray unit operating at 85 kVp, 9.6 mA, dose rate $\sim0.95\,Gy\,min^{-1}$.

### *Drug exposure*

Lonidamine was made up in a stock solution with

© The Macmillan Press Ltd., 1984

dimethyl sulfoxide (DMSO) as the solvent. At the highest lonidamine concentration used, $50\,mg\,l^{-1}$, the medium contained 1% DMSO. For this reason, 1% DMSO controls, without lonidamine, were always included. Lonidamine so dissolved was non-toxic to cells for exposures of $50\,mg\,l^{-1}$ and for up to 24 h. Methyl methane sulfonate (MMS), at a concentration of $600\,mg\,l^{-1}$ was dissolved in MEM not containing serum. Bleomycin was used at a concentration of $50\,mg\,l^{-1}$ and it was also dissolved in MEM without serum. In experiments requiring drug removals or exchanges, cells were washed twice in MEM without serum before new medium containing different drugs, or no drugs, was added to the dishes.

### Results

### *X-irradiation*

Several types of experiments were performed to examine the ability of the drug to interfere with the recovery from PLD. We tested the need for the agent to be present during, before or after X-ray. Drug present only before X-ray had no effect on survival. Results of the other experiments are shown in Figures 1–3. The first of these shows that doses up to $25\,mg\,l^{-1}$ are unable to abolish recovery if the drug is present only before and during X-irradiation. The second figure, however, shows that if lonidamine is present after irradiation,

**Figure 1** Survival of plateau phase HA-1 cells after X-irradiation: lonidamine before and during X-ray. Cells were incubated in Hank's Balanced Salt Solution (HBSS) with lonidamine (concentration as indicated) for 16 h before being given 12-Gy of 80 kVp X-rays. Immediately after irradiation, one group of cells was trypsinized and plated for colony formation. The other 2 groups were rinsed and incubated in HBSS without lonidamine for either 3 or 6 h and then trypsinized. (○) no lonidamine; (□) $10\,mg\,l^{-1}$ lonidamine; (△) $25\,mg\,l^{-1}$ lonidamine.

**Figure 2** Survival of plateau phase HA-1 cells after X-irradiation: lonidamine after X-rays. Cells were given 12-Gy and then either trypsinized and plated for colony formation or reincubated in lonidamine in HBSS at the indicated concentrations. (○) no lonidamine; (△) $10\,mg\,l^{-1}$ lonidamine; (□) $50\,mg\,l^{-1}$ lonidamine.

**Figure 3** Survival of plateau phase HA-1 cells after X-irradiation: lonidamine before, during and after X-ray. Protocol as in **Figure 1**, except that cells were maintained in HBSS with lonidamine until trypsinization at the indicated times. (○) no lonidamine; (△) $10\,mg\,l^{-1}$ lonidamine.

it inhibits recovery. At a dose of $50\,mg\,l^{-1}$, recovery is completely inhibited. Finally, in Figure 3 we show results of an experiment in which the drug was present before, during and after irradiation. Under such conditions the presence in the medium of $10\,mg\,l^{-1}$ is sufficient to completely inhibit any increase in survival. Higher doses, although by themselves completely non-toxic, increase the radiation-induced cell lethality below control values, thus favoring "fixation" of X-ray damage.

*Methyl methane sulfonate*

Figure 4 shows results of a very similar experiment to the one just described, but using MMS as the cytotoxic agent. Again the ability of lonidamine to interfere with recovery is clearly seen. Where lonidamine was present both before and after MMS exposure, additional inhibition of recovery was seen.

**Figure 4** Survival of plateau phase HA-1 cells after MMS exposure: lonidamine after drug exposure. Cells were exposed to MMS (20 min, $600 mg l^{-1}$) and then either plated for colony formation or incubated in HBSS with lonidamine until trypsinization at the indicated times. One group of cells also had lonidamine ($25 mg l^{-1}$) present for 16 h before as well as after drug exposure. (○) no lonidamine; (△) $10 mg l^{-1}$ lonidamine; (□) $25 mg l^{-1}$ lonidamine. (◇) $50 mg l^{-1}$ lonidamine. (▽) $25 mg l^{-1}$ lonidamine before and after MMS exposure.

*Bleomycin*

HA-1 cells are relatively resistant to this drug. Its cytotoxicity is, however, greatly enhanced at elevated temperatures (Braun & Hahn, 1975). We therefore exposed cells for 40 min at 43°C followed by 1 h bleomycin treatment and then tested their ability to recover from PLD at 37°C. Results are shown in Figure 5. Again lonidamine's ability to interfere with recovery is apparent, although the magnitude of the effect is smaller than after either X-irradiation or MMS. When lonidamine was present before and after bleomycin and heat, a small additional amount of inhibition was seen (data not shown).

Twentyman & Bleehen (1975) have suggested that there is no recovery from PLD after bleomycin treatment, but only a time-dependent disappearance of a trypsin-bleomycin interaction. Specifically, they suggest that trypsin permits membrane-bound drug to enter the cell. If this were the case, our data on bleomycin might have no clinical relevance. While

**Figure 5** Survival of plateau phase HA-1 cells after bleomycin and heat: lonidamine after treatment. Cells were first heated at 43°C for 40 min; this was followed by bleomycin ($50 mg l^{-1}$, 1 h). They were then either plated for colony formation or incubated at 37°C in HBSS with or without lonidamine until trypsinization at the indicated times. (○) no lonidamine; (△) $25 mg l^{-1}$ lonidamine.

**Figure 6** Repair of potentially lethal damage after heat and bleomycin exposures. Cells were trypsinized, diluted, plated at appropriate concentrations and allowed to attach for 6 h. They were then rinsed twice with medium and then returned to the 37°C incubator for an additional 12 h. Then they were heated (43°C, 40 min) and exposed to bleomycin ($50 mg l^{-1}$; 1 h). At the indicated times, the medium was replaced with hypotonic phosphate-buffered saline (0.63M PBS). Twenty minutes later, the buffer was removed, medium added and the cells placed in the incubator for colony formation. An additional heat only control showed no effects of the hypotonic buffer treatment. In another experiment, hypertonic phosphate-buffered saline (1.83 M PBS) was used; results were indistinguishable from those shown.

the results presented in Figure 5 are difficult to rationalize on that basis, nevertheless we performed experiments to test for PLD recovery in a system that does not involve trypsinization. We utilized the technique of interrupting recovery by exposing cells to a non-isotonic environment (Utsumi & Elkind, 1979). Results for hypotonic exposures are shown in Figure 6. Clearly, recovery did occur, even though in these experiments no trypsinisation was involved. In another experiment we used hypertonic medium; results were similar to those presented.

### Discussion

The data presented here show lonidamine can inhibit recovery from PLD caused by X-rays, MMS, or by bleomycin and heat. These agents were chosen because earlier studies had shown that PLD recovery from exposures could be demonstrated in the plateau phase system. We have no data that can be used to determine how lonidamine accomplishes this. Some clue may be obtained, however, by considering the drug's mode of action as a cytotoxic agent. Lonidamine is said to act against cells by interfering with the integrity of condensed mitochondria, and thereby presumably disrupting energy metabolism. It is a reasonable hypothesis to suggest that this is also an explanation for its anti-recovery action because Jain *et al.* (1982) have recently demonstrated that, at least in X-irradiated yeast, recovery from PLD is an energy-requiring process. This hypothesis is certainly consistent with our finding that lonidamine is most effective when it is present in the cultures both before and after irradiation.

Weichselbaum *et al.* (1977) have presented data which argue that the radiation resistance of some tumours, particularly melanomas, may be related to an unusual capacity of cells from such tumours to deal with PLD. If this is correct, then the chronic administration of lonidamine during a fractionated course of radiotherapy might well make such lesions much more responsive to X-rays. Any possible therapeutic effect would also have to take into account PLD recovery in normal tissues. Our results also suggest that the drug may be of use during poly-drug chemotherapy.

An advantageous aspect of our finding is that the toxicity and pharmaco-kinetics of this drug are already being studied extensively. Lonidamine is currently undergoing phase I and II studies at several institutions, both in the U.S. and in Canada, as well as in Italy, for possible anti-tumour activity. Plasma levels of $10\,mg\,l^{-1}$ and even higher have been found to be readily achievable and are apparently not accompanied by undue toxicities. Therefore this drug may be suitable for clinical testing in the near future.

### References

BRAUN J. & HAHN, G.M. (1975). Enhanced cell killing by bleomycin and 43°C hyperthermia and the inhibition of recovery from potentially lethal damage. *Cancer Res.*, **35**, 2921.

EVANS, R.G., BAGSHAW, M.A., GORDON, L.F., KURKJIAN, S.D. & HAHN, G.M. (1974). Modification of recovery from potentially lethal X-ray damage in plateau phase Chinese hamster cells. *Radiat. Res.*, **59**, 597.

GUICHARD, M., DE LANSEN-ORMI, F. & MALAISE, E.P. (1979). Influence of misonidazole on the radiosensitivity of human melanoma in nude mice: time dependent increases in surviving fraction. *Int. J. Radiat. Oncol. Biol. Phys.*, **5**, 487.

HAHN, G.M. (1976). Recovery of cells from induced, potentially lethal damage. *Cancer Treat. Rep.*, **60**, 1791.

HAHN, G.M. & LITTLE, J.B. (1972). Plateau phase cultures of mammalian cells in an *in vitro* model for human cancer. *Curr. Topics Radiat. Res. Quart.*, **8**, 39.

ILLIAKIS, G. (1980). Effects of B-arabinofuranosyladenine on the growth and repair of potentially lethal damage in Erlich Ascites tumor cells. *Radiat. Res.*, **83**, 537.

JAIN, V.K., GUPTA, I. & LATA, K. (1982). Energetics of cellular repair processes in a respiratory deficient mutant of yeast. *Radiat. Res.*, **92**, 474.

PHILLIPS, R.A. & TOLMACH, L.S. (1966). Repair of potentially lethal damage in X-irradiated HeLa Cells. *Radiat. Res.*, **29**, 413.

TWENTYMAN, P.R. & BLEEHEN, N.M. (1975). Studies of potentially lethal damage in EMT6 mouse tumor cells heated with bleomycin either *in vitro* or *in vivo*. *Br. J. Cancer*, **32**, 491.

UTSUMI, H. & ELKIND, M.M. (1979). Potentially lethal damage versus sublethal damage: Independent repair processes in actively growing Chinese hamster cells. *Radiat. Res.*, **77**, 346.

WEICHSELBAUM, R.R., LITTLE, J.B. & NOVE, J. (1977). Response of osteosarcoma *in vitro* to X-irradiation. Evidence for unusual cellular repair activity. *Int. J. Radiat. Biol.* **31**, 295.